# CircLMTK2 Silencing Attenuates Gemcitabine Resistance in Pancreatic Cancer by Sponging miR-485-5p and to Target PAK1

**DOI:** 10.1155/2022/1911592

**Published:** 2022-08-24

**Authors:** Yeting Lu, Shuping Zhou, Gong Cheng, Yi Ruan, Yuan Tian, Kaiji Lv, Shuo Han, Xinhua Zhou

**Affiliations:** ^1^Department of General Surgery, Ningbo Medical Center Lihuili Hospital, The Affiliated Lihuili Hospital, Ningbo University, Ningbo 315100, Zhejiang, China; ^2^Ningbo College of Health Sciences, Ningbo 315100, Zhejiang, China; ^3^Department of Healthcare Security and Price Management, Ningbo Medical Center Lihuili Hospital, The Affiliated Lihuili Hospital, Ningbo University, Ningbo 315100, Zhejiang, China

## Abstract

Pancreatic cancer (PC) has a high degree of malignancy and poor prognosis, and countless patients have distant metastasis when diagnosed. Gemcitabine (GEM) chemotherapy is one of the main ways of treatment. However, PC cells have been displayed chemoresistance to GEM during treatment. Circular RNAs (circRNAs) have been demonstrated to be the most popular diagnostic and prognostic biomarkers in PC with GEM resistance. Here, we assessed the potential of circLMTK2 in the GEM resistance of PC cells. Functional assays were implemented to measure the impacts of circLMTK2 on the proliferation, migration/invasion, and apoptosis of GEM-resistant PC cells. Bioinformatics analysis and mechanical experiments displayed the underlying mechanism of circLMTK2 in GEM-resistant PC cells. We found that circLMTK2 was upregulated in PC and GEM-resistant PC tissues and cells. CircLMTK2 knockdown suppressed proliferation, invasion, migration, and enhanced apoptosis in GEM-resistant PC cells. Moreover, circLMTK2 silencing could decrease GEM resistance-associated tumor size *in vivo*. In terms of mechanism, circLMTK2 served as a sponge for miR-485-5p, and miR-485-5p bound to p21 (RAC1) activated kinase 1 (PAK1), which were clarified via the dual-luciferase assay in PC cell lines. We confirmed that circLMTK2 knockdown attenuated GEM-resistant PC cells by regulating PAK1 via miR-485-5p. Our study demonstrated that circLMTK2 may be a novel diagnostic and prognostic biomarker in GEM-resistant PC cells.

## 1. Introduction

Pancreatic cancer (PC), a malignant tumor with a high degree of malignancy and poor prognosis, has a 5-year survival rate less than 7% [1]. Numerous patients have been diagnosed with distant metastasis and thus lost the chance of surgery [2]. Therefore, chemotherapy is considered to be the main treatment for improving the prognosis of patients with PC. Gemcitabine (GEM) is a first-line treatment for locally advanced and metastatic PC [3]. However, GEM resistance is an important factor limiting the survival time of patients with PC [4]. Therefore, it is of great importance to investigate the molecular mechanism of GEM resistance in PC and explore the key regulatory targets that mediate GEM resistance.

Although many signaling pathways and downstream genes have been confirmed to be involved in the regulation of GEM resistance in PC, such as PI3K/AKT, Wnt, ERK, HMGB1, MDR1, and so on [5–8]. However, the regulatory mechanism of GEM resistance in PC is still unclear. The occurrence of chemoresistance in PC is related to the imbalance of key gene expression and epigenetic regulation network [9]. CircRNAs are a novel class of noncoding RNAs that are circularized by connecting the 3′-5′. Studies have confirmed that circRNAs have the advantages of stable structure, high conservation, and tissue-specific expression [10]. Various studies have shown that circRNAs, as an important part of gene regulatory network and epigenetic regulatory network, play important roles in chemoresistance in cancer therapy [11, 12]. For instance, circ_0058063 could regulate the cisplatin-resistance of bladder cancer cells via the miR-335-5p/B2M axis [13]. Besides, high circFARP1 levels were confirmed positively related to elevated serum LIF levels and poor survival in pancreatic ductal adenocarcinoma. Silencing circFARP1 expression could suppress pancreatic ductal adenocarcinoma growth and GEM resistance [14]. Therefore, further studies on the role and mechanism of circRNAs in chemotherapy resistance of PC are important.

In solid tumors, circRNA mainly enhances or inhibits the expression of its target genes by binding with microRNAs (miRNAs), and regulates the metabolic functions of cell proliferation, differentiation, and apoptosis [15]. Based on the above research background, we first systematically analyzed the GEM resistance-related circRNAs/miRNA/mRNA network in PC using bioinformatics analysis. Through interaction network analysis and previous study results, the circLMTK2-miR-485-5p-PAK1 axis attracted our attention.

In this study, we detected the expression of circLMTK2 in PC and evaluate its impact on prognosis in PC patients. We also explored the regulatory effects of circLMTK2 on the proliferation, apoptosis, invasion, and migration in GEM-resistant PC cells. In addition, we investigated the effect of circLMTK2 on GEM-resistant tumor growth in vivo. Finally, we clarified the potential molecular mechanism of circLMTK2 in GEM-resistant PC cells.

## 2. Methods

### 2.1. Patients

The study was approved by the Ethics Committee of the Lihuili Hospital affiliated to Ningbo University and obtained the written informed consent of all subjects. The cancer tissues and adjacent tissues of 40 patients with PC were obtained from the Lihuili Hospital affiliated to Ningbo University during surgery. GEM-resistant: PC patients had no effect on GEM treatment, or the tumor progressed after previous GEM treatment (no more than 6 months).

### 2.2. Cell Culture and Transfection

Human PC cells (MIA-PaCa-2, PANC-1, BxPC-3, and SW 1990) were obtained from Cell Bank of Chinese Academy of Sciences (Shanghai, China). MIA-PaCa-2 and PANC-1 cells were maintained in Dulbecco's Modified Eagle's Medium. BxPC-3 cells were maintained in RPMI-1640 medium. SW 1990 cells were maintained in Leibovitz's L-15 medium. All cell mediums were cultured in a 5% CO_2_ incubator at 37°C. PANC-1-GEM and MIA-PaCa-2-GEM were created according by previous study [16]. sh-circLMTK2, miR-485-5p mimic/inhibitor, sh-PAK1/pcDNA3.1 PAK1, or negative control (NC) were transfected into PANC-1-GEM and MIA-PaCa-2-GEM cells using Lipo 3000 (Thermo Fisher Scientific, USA) according to the manufacture's protocol.

### 2.3. Real-Time Quantitative PCR Analysis

Total RNA from samples was extracted using TRizol reagent (Thermo Fisher Scientific, USA), and was further reverse transcribed into cDNA. Fast SYBR Green Master Mix (Thermo Fisher Scientific, USA) was then used to carry out qRT-PCR on Real-Time System (Bio-Rad, USA). Each sample was set to 3 repetitions; the expressions of circLMTK2, miR-485-5p, and PAK1 were analyzed using the 2^−ΔΔCT^ method. Primer information is provided in Table 1.

### 2.4. Western Blot Analysis

The protein was extracted using lysis buffer, and protein concentration was measured via BCA assay kits. After electrophoresis and membrane transfer, the bands were incubated with the primary antibody including PAK1 (1/1000, ab223849, Abcam) and GAPDH (1/2500, ab9485, Abcam) at 4°C overnight, then cultured with secondary antibody at room temperature for 2 h. The chemiluminescence system (Bio-Rad, USA) imaging system was performed to measure all bands.

### 2.5. Subcellular Localization Assay

Nuclear, cytoplasmic, and total RNA was isolated using a PARIS™ kit (Invitrogen), followed by qRT-PCR analysis. U6 was used as endogenous controls for the nucleus, while GAPDH was used for the cytoplasm.

### 2.6. Cell Counting Kit-8 (CCK-8) Assay

Cells were seeded in a 96-well plate for 24 h and were transfected with the indicated plasmids. Ten microliters of CCK-8 solution (Beyotime, Shanghai, China) was then added into each well for 2 h at 37°C. The absorbency was measured at 450 nm by using a microplate reader.

### 2.7. Wound Healing and Transwell Assay

The adherent cells were scraped vertically using the 200-*μ*L pipette and cultured with the medium containing 1% FBS. The wound healing was confirmed under an inverted microscope (Zeiss, Japan) at 0 and 24 hours, and photos were taken. In the transwell assay, total 1 × 10^4^ transfected cells were added and cultured on the upper chamber with Matrigel (BD Bioscience, CA, USA) coated, and the bottom chamber was seeded 500 *μ*L medium with 10% FBS. After 48 h incubation, the upper chamber was removed and the bottom chamber of cells was fixed using formaldehyde. The invaded cell number was counted via an inverted microscope (Zeiss, Japan).

### 2.8. Terminal deoxynucleotidyl transferase-mediateddUTP-nick end labeling (TUNEL)

Based on the instructions, cell apoptosis was detected by the TUNEL detection kit (Vazyme).

### 2.9. Luciferase Reporter Assay

First, construction of miR-485-5p and circLMTK2 wild-type (WT) or circLMTK2 mutant-type (MUT) plasmids, miR-485-5p, and PAK1 wild-type (WT) or PAK1 mutant-type (MUT) plasmids were performed. The recombinant plasmid was then transfected into PC cells with miR-485-5p mimic or mimic NC. After 48 h, luciferase activities were measured using Dual-Luciferase Reporter Assay System (Promega) according to manufacturer's protocol.

### 2.10. Mouse Xenograft Assay

Total 100 *μ*l of sh-circLMTK2 or sh-NCGEM-resistant PANC-1 cells (2 × 10^7^/mL) were injected into the abdominal cavity of BALB/c nude mice. The tumor formation could be touched after 1 week, and then the tumor size was measured every week, and the tumor body was cut and weighed at the fourth week. The animal experiments were approved by the Institutional Animal Care and Use Committee of Lihuili Hospital affiliated to Ningbo University and were implemented following the institutional guidelines.

### 2.11. Statistical Analysis

The SPSS 22.0 software was conducted for data analysis in this study. Mean ± SD was used to represent the measurement data. Student's *t*-test was performed to analyze statistical analysis between two groups. ANOVAs with Bonferroni post hoc tests were used for more than three groups. The survival rate was analyzed by Kaplan–Meier curve. *P* < 0.05 was considered statistical significance.

## 3. Results

### 3.1. circLMTK2 is Increased in PC and GEM-Resistant PC Tissues and Cells

**Figure 1:**
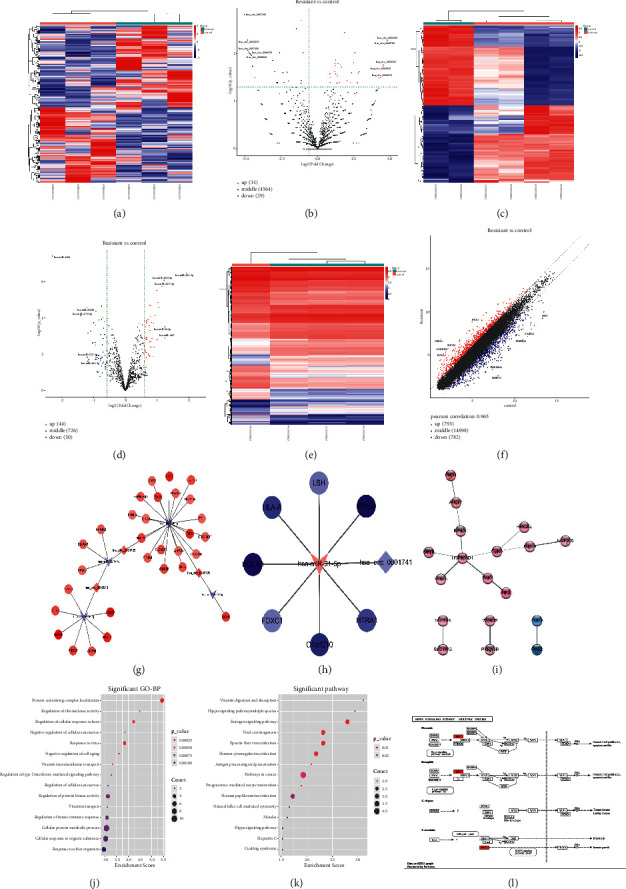
Identification of circRNA, miRNA, and mRNA. (a–f) The heat map and volcano map or scatter plot of genes with significant differences. (g–h) circRNA-miRNA-mRNA interaction network of the upregulated and downregulated circRNAs. (i) PPI interaction network of the significantly different genes located in the two ceRNA networks. (j) The enriched GO terms of the differentially expressed genes. (k) The enriched KEGG pathways of the differentially expressed genes. (l) The differential genes were mapped in significantly enriched pathways using Pathview package.

From the GEO database, we analyzed the GSE110580 dataset, the GSE79234 dataset, and the GSE80617 dataset. The screening threshold for significant differences in gene expression was *P* < 0.05 and |log2fc| > 0.585, and the heat map and volcano map or scatter plot of genes with significant differences in this analysis were as follows (Figure 1(a)–1(f)).

After obtaining significantly different circRNA, miRNA, and mRNA, we tried to construct a circRNA-miRNA-mRNA interaction network which is displayed in Figures 1(g)-1(h). For the significantly different genes located in the two ceRNA networks, the protein-protein interaction (PPI) interaction network was analyzed by string database and visualized by Cytoscape software (Figure 1(i)). The enriched gene ontology (GO) terms and enriched Kyoto Encyclopedia of Genes and Genomes (KEGG) pathways of the differentially expressed genes are displayed in Figures 1(j) and 1(k). Finally, the differential genes were mapped in significantly enriched pathways using Pathview package (Figure 1(l)).

**Figure 2:**
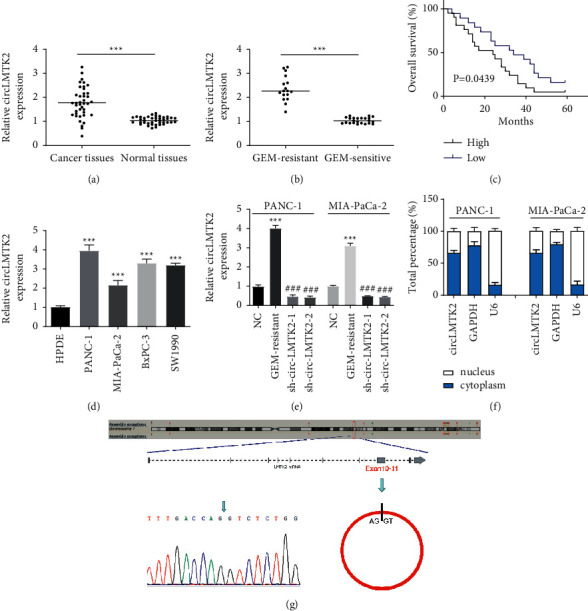
CircLMTK2 is increased in PC and GEM-resistant PC tissues and cells. (a, b) circLMTK2 expression in PC and GEM-resistant PC tissues was determined by the qRT-PCR assay. (c) The relationship between circLMTK2 expression and survival rate was estimated using Kaplan–Meier analysis. (d) circLMTK2 expression in PC cell lines was determined by the qRT-PCR assay. (e) circLMTK2 expression in GEM-resistant PC cell lines and the success of knockdown of circLMTK2 with sh-circLMTK2-1/2 were confirmed by qRT-PCR analysis. (f) The amplified product was sequenced using Sanger sequencing to validate the circularized junction of circLMTK2. (g) The location of circLMTK2 was verified by the subcellular fractionation assay. ^*∗∗∗*^*P* < 0.001 vs. NC group; ^###^*P* < 0.001 vs. GEM-resistant group.

Considering that PAK1 was abnormally expressed in pancreatic cancer and could inhibit DNA damage and inhibit cell apoptosis via the NF-*κ*B pathway, thereby making pancreatic cancer cells resistant to GEM in the previous study, we hypothesized that circLMTK2 might regulate the expression of PAK1 by binding to miR-485-5p through the circRNA-miRNA-mRNA interaction network. Thus, we used qRT-PCR to investigate the circLMTK2 expression in human PC and GEM-resistant PC tissues. The results indicated that circLMTK2 was significantly increased in PC and GEM-resistant PC tissues (Figure 2(a) and 2(b)). Besides, the Kaplan–Meier survival analysis displayed that higher circLMTK2 expression led to poorer survival rate (Figure 2(c)). Interestingly, we also found that circLMTK2 expression was increased in PC cell lines (Figure 2(d)). PANC-1 and MIA-PaCa-2 cell lines were selected for the following study due that both cell lines harbored higher circLMTK2 expression than other PC cell lines. After creating successful PANC-1 and MIA-PaCa-2-GEM-resistant cells, we detected circLMTK2 expression was further increased in GEM-resistant PC cell lines (Figure 2(e)). In addition, the amplified product was sequenced using Sanger sequencing to validate the circularized junction of circLMTK2 (Figure 2(f)). To study the location of circLMTK2, we conducted the subcellular fractionation assay. The result indicated that circLMTK2 was mainly expressed in cytoplasm (Figure 2(g)).

### 3.2. circLMTK2 Knockdown Suppresses Proliferation, Invasion, and Migration, and Enhances Apoptosis in GEM-Resistant PC Cells

**Figure 3:**
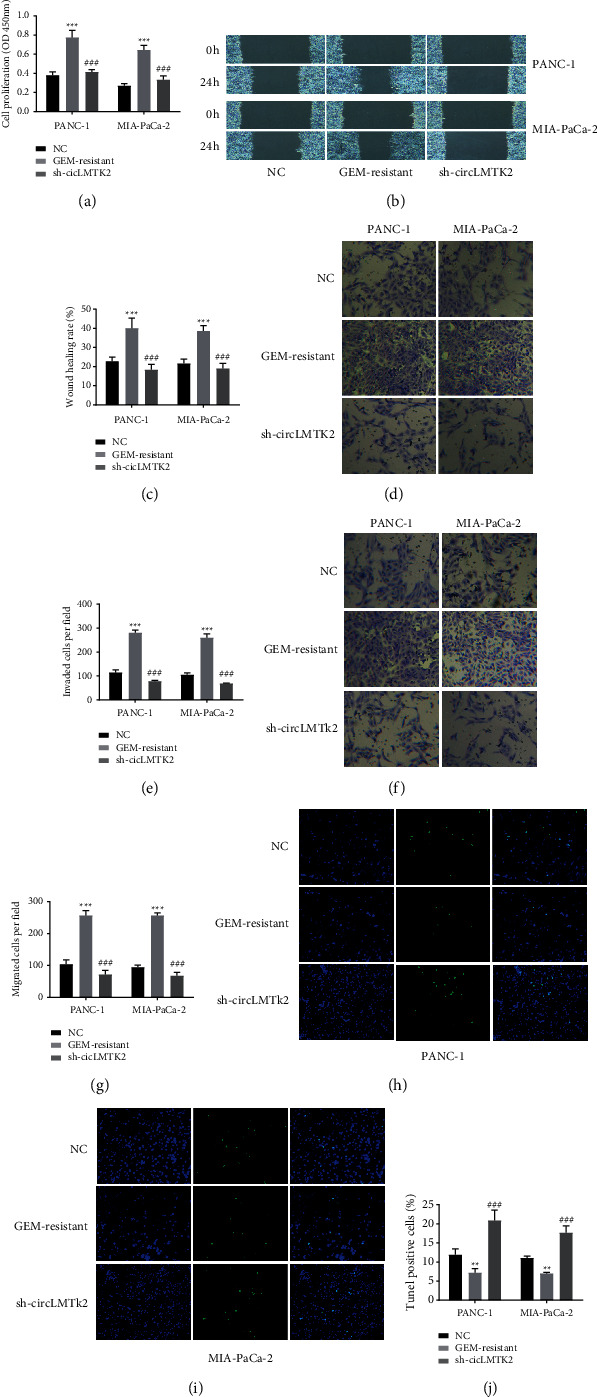
CircLMTK2 knockdown suppresses proliferation, invasion, and migration, and enhances apoptosis in GEM-resistant PC cells. (a) CCK-8 assay was used to determine cell proliferation. (b–g) Wound healing and transwell assays were carried out to detect cell invasion and migration. (h–j) The TUNEL assay was used to determine cell apoptosis. ^*∗∗*^*P* < 0.01; ^*∗∗∗*^*P* < 0.001 vs. NC group; ^###^*P* < 0.001 vs. GEM-resistant group.

To study the function of circLMTK2 in GEM-resistant PC cells, we first successfully inhibited circLMTK2 expression via transfecting sh-circLMTK2-1 and sh-circLMTK2-2 into GEM-resistant PC cells. The CCK-8 assay demonstrated that the knockdown of circLMTK2 significantly attenuated cell proliferation in GEM-resistant PC cells (Figure 3(a)). Besides, wound healing and transwell assays indicated circLMTK2 knockdown could significantly suppress cell migration and invasion in GEM-resistant PC cells (Figure 3(b)–3(g)). In addition, the knockdown of circLMTK2 promoted GEM-resistant PC cells' apoptosis as exhibited by the Tunel assay (Figure 3(h)–3(j)).

### 3.3. circLMTK2 Directly Binds to miR-485-5p

**Figure 4:**
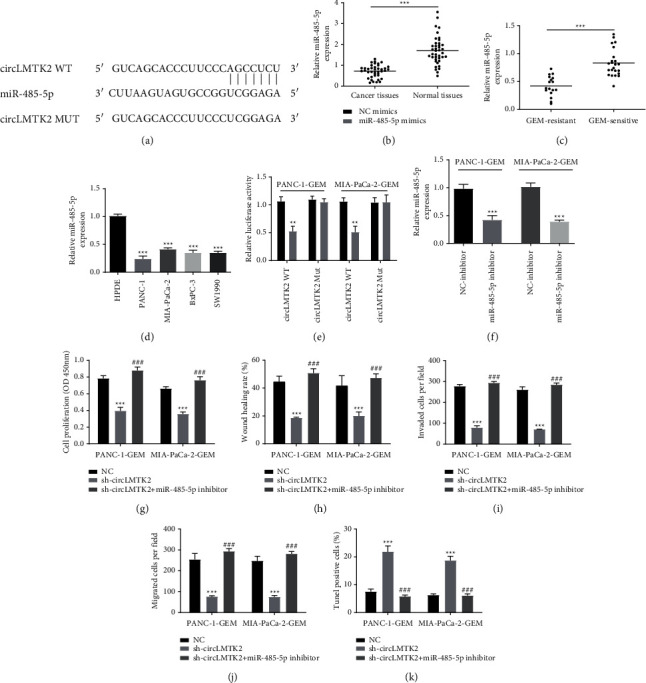
circLMTK2 is directly bound to miR-485-5p. (a) The binding sites of miR-485-5p in circLMTK2. (b–d) The miR-485-5p expressions in PC and GEM-resistant PC tissues and cell lines were determined by the qRT-PCR assay. (e) The luciferase reporter assay was used to determine the physical connection between circLMTK2 and miR-485-5p. (f) The success of miR-485-5p inhibition with miR-485-5p inhibitor was confirmed by qRT-PCR analysis. (g) The CCK-8 assay was used to determine cell proliferation. (h–j) Wound healing and transwell assays were carried out to detect cell invasion and migration. (k) The TUNEL assay was used to determine cell apoptosis. ^*∗∗*^*P* < 0.01; ^*∗∗∗*^*P* < 0.001 vs. NC group; ^###^*P* < 0.001 vs. sh-circLMTK2 group.

After then, the binding sites of miR-485-5p in circLMTK2 were predicted and mutated accordingly (Figure 4(a)), and we found miR-485-5p was downregulated significant in PC and GEM-resistant PC tissues, as well as PC cell lines (Figure 4(b)–4(d)). Then, the luciferase reporter assay was used to determine the physical connection between circLMTK2 and miR-485-5p in PANC-1 and MIA-PaCa-2 GEM-resistant cells. We found that miR-485-5p mimics reduced the luciferase activity of circLMTK2 wild-type but had no impacts on that of circLMTK2 mutant sites (Figure 4(e)).

To investigate the role of miR-485-5p in PANC-1 and MIA-PaCa-2 GEM-resistant cells, we tried to knock down miR-485-5p expression via miR-485-5p inhibitor (Figure 4(f)). Interestingly, the CCK-8 assay indicated miR-485-5p inhibitor could reverse the antiproliferation effect of sh-circLMTK2 (Figure 4(g)). Besides, the suppression of migration and invasion of PANC-1 and MIA-PaCa-2 GEM-resistant cells induced by sh-circLMTK2 could be revised by miR-485-5p inhibitor (Figure 4(h)–4(j)). In addition, the Tunel assay showed that miR-485-5p inhibition could restore the enhanced apoptosis caused by silencing circLMTK2 (Figure 4(k)).

### 3.4. PAK1 is a Direct Target of miR-485-5p

**Figure 5:**
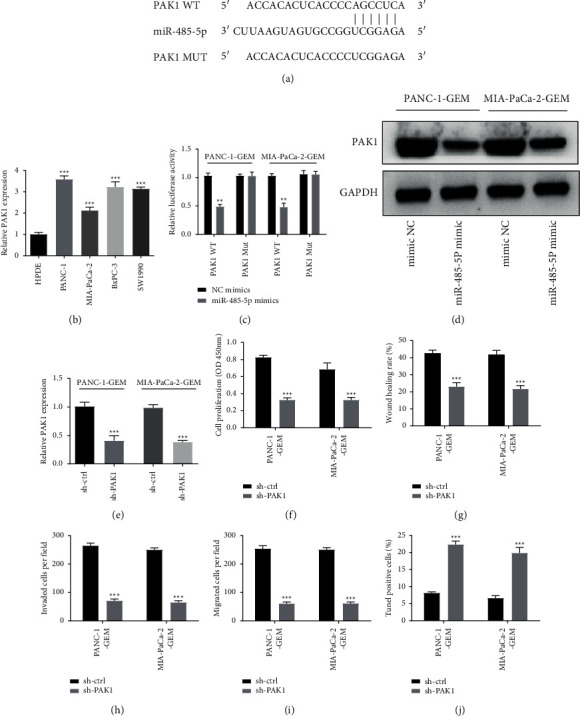
PAK1 is a direct target of miR-485-5p. (a) The binding site between miR-485-5p and the 3′-UTR of PAK1. (b) The PAK1 expression in PC cell lines was determined by the qRT-PCR assay. (c) The luciferase reporter assay was conducted to investigate the connection between miR-485-5p and PAK1. (d) Western blot analysis was used to examine the PAK1 expression. (e) The success of PAK1 inhibition with sh-PAK1 was confirmed by qRT-PCR analysis. (f) The CCK-8 assay was used to determine cell proliferation. (g–i) Wound healing and transwell assays were carried out to detect cell invasion and migration. (j) The TUNEL assay was used to determine cell apoptosis. ^*∗∗*^*P* < 0.01; ^*∗∗∗*^*P* < 0.001.

Subsequently, the binding site between miR-485-5p and the 3′-UTR of PAK1 is shown in Figure 5(a). Interestingly, PAK1 expression was elevated in PC cell lines (Figure 5(b)). Then, the luciferase reporter assay was used to determine the physical connection between PAK1 and miR-485-5p in PANC-1 and MIA-PaCa-2 GEM-resistant cells. We found that miR-485-5p mimics reduced the luciferase activity of PAK1 wild-type but had no impacts on that of PAK1 mutant sites (Figure 5(c)). We next treated PANC-1 and MIA-PaCa-2 cells with miR-485-5p mimic and found that the expression of PAK1 was significantly downregulated in miR-485-5p mimic groups than mimic NC groups (Figure 5(d)). To investigate the role of PAK1 in PANC-1 and MIA-PaCa-2 GEM-resistant cells, we tried to inhibit PAK1 via transfecting with sh-PAK1 (Figure 5(e)). The CCK-8 assay indicated that PAK1 inhibition significantly weakened cell proliferation in PANC-1 and MIA-PaCa-2 GEM-resistant cells (Figure 5(f)). In addition, silencing PAK1 could suppress the migration and invasion of PANC-1 and MIA-PaCa-2 GEM-resistant cells (Figure 5(g)–5(i)). Moreover, the TUNEL assay demonstrated that knockdown of PAK1 could enhance apoptosis in PANC-1 and MIA-PaCa-2 GEM-resistant cells (Figure 5(j)).

### 3.5. PAK1 Overexpression Eliminates the Effect of circLMTK2 Knockdown

**Figure 6:**
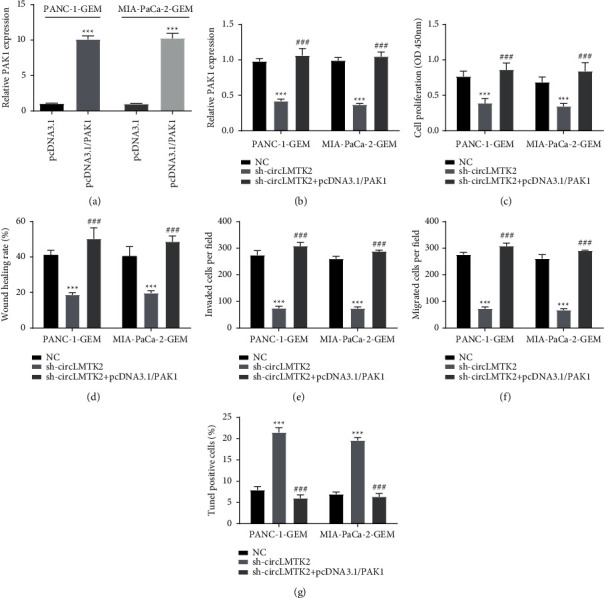
PAK1 overexpression eliminates the effect of circLMTK2 knockdown. (a) The efficiency of pcDNA3.1/PAK1 was confirmed by the qRT-PCR assay. (b) The PAK1 expression was evaluated by the qRT-PCR assay. (c) The CCK-8 assay was used to determine cell proliferation. (d–f) Wound healing and transwell assays were carried out to detect cell invasion and migration. (g) The TUNEL assay was used to determine cell apoptosis. ^*∗∗*^*P* < 0.01; ^*∗∗∗*^*P* < 0.001 vs. NC group; ^###^*P* < 0.001 vs. sh-circLMTK2 group.

Next, we verified whether circLMTK2 exerted its role in PANC-1 and MIA-PaCa-2 GEM-resistant cells via the miR-485-5p/PAK1 pathway through functional rescue experiment. The qRT-PCR assay was used to confirm the efficiency of pcDNA3.1/PAK1, and the inhibitory effect of silencing circLMTK2 on PAK1 expression could be revised by pcDNA3.1/PAK1 transfection (Figure 6(a) and 6(b)). The CCK-8 assay showed that cell proliferation attenuated via sh-circLMTK2 could be restored by PAK1 overexpression (Figure 6(c)). Besides, the suppression of migration and invasion of PANC-1 and MIA-PaCa-2 GEM-resistant cells induced by sh-circLMTK2 could be revised by PAK1 overexpression (Figure 6(d)–6(f)). In addition, the TUNEL assay showed that PAK1 overexpression could restore the enhanced apoptosis caused by silencing circLMTK2 (Figure 6(g)).

### 3.6. circLMTK2 Knockdown Inhibits the Growth of PC Tumor *In Vivo*

**Figure 7:**
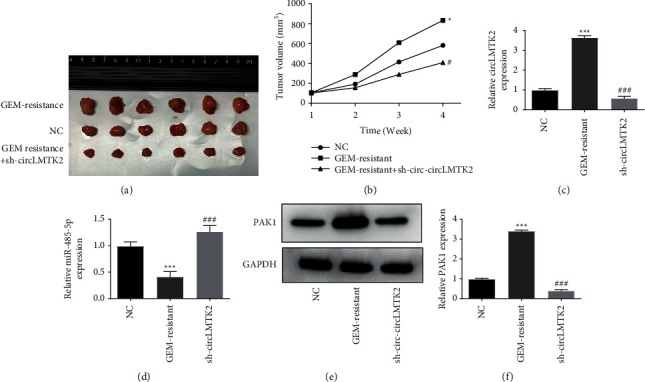
circLMTK2 knockdown inhibits the growth of PC tumor *in vivo*. (a-b) The volume of tumors was recorded. (c-d) The mRNA expression levels of circLMTK2 and miR-485-5p were determined in tumor tissues. (f) The protein expression of PAK1 were determined in tumor tissues. ^*∗∗*^*P* < 0.01; ^*∗∗∗*^*P* < 0.001 vs. NC group; ^###^*P* < 0.001 vs. GEM-resistant group.

Finally, to investigate the role of circLMTK2 in PC *in vivo*, we established the xenograft model by injecting PANC-1-GEM-resistant cells transfected with sh-circLMTK2 or sh-NC into nude mice. The results indicated that the tumor size and tumor volume in the sh-circLMTK2 group were decreased significantly than that in sh-NC groups (Figure 7(a) and 7(b)). In addition, the circLMTK2 and PAK1 expression were decreased while the miR-485-5p expression was increased in tumor tissues in the sh-circLMTK2 group compared with those in the sh-NC group, respectively (Figure 7(c)–7(f)).

## 4. Discussion

Although GEM has been considered as the first-line therapeutic agent for pancreatic cancer, the drug resistance of GEM has become an important factor affecting the chemotherapy effect of pancreatic cancer [17, 18]. Using bioinformatics analysis, we analyzed previous high throughput datasets of GEM resistance, revealed their network relationships, and obtained the circLMTK2/miR-485-5p/PAK1 axis that might play a regulatory role in pancreatic cancer.

Studies have shown that the dysregulated expression of circRNAs in tumors can not only be used as biological markers for diagnosis and prognosis but also as potential intervention targets for tumor treatment and chemoresistance. For example, circBFAR was upregulated in pancreatic ductal adenocarcinoma, and the ectopic expression of circBFAR was positively associated with poorer prognosis of pancreatic ductal adenocarcinoma patients [19]. Similarly, circRNAs are also playing important roles in the field of chemotherapy resistance, Liu et al. revealed that circHIPK3 could enhance GEM resistance in PC cells through targeting RASSF1 via sponging miR-330-5p [20]. In this study, we confirmed that circLMTK2 was significantly increased in PC and GEM-resistant PC tissues. Kaplan–Meier survival analysis showed that circLMTK2 expression was significantly negatively correlated with survival. Furthermore, circLMTK2 knockdown could inhibit cell proliferation, invasion/migration, and enhances apoptosis in GEM-resistant PC cells. Our study suggests that elevated expression of circLMTK2 is an independent risk factor for poor prognosis of PC and may be a prognostic marker for PC, and a potential therapeutic target for GEM chemotherapy in PC.

In terms of the regulatory mechanism of circRNAs on tumor cell proliferation, differentiation, and chemoresistance, a large number of studies have confirmed that circRNAs can affect their transcription and translation by sponging miRNAs, and then regulate the expression of related tumor genes, leading the occurrence and development of cancers through various related signal transduction pathways [21]. In this study, biological information analysis and experimental verification reveal that miR-485-5p bound to circLMTK2. miR-485-5p has been confirmed to play an important role in the development of cancer in previous studies [22, 23]. However, the function role of miR-485-5p in PC is still unknown. Through validation, we found that circLMTK2 could negatively regulate the expression of miR-485-5p, thereby mediating the proliferation, invasion/migration, and apoptosis of PC cells. Next, we further revealed the downstream target PAK1 of the circLMTK2/miR-485-5p axis, which has been proved to be an important therapeutic target for PC [24]. PAK1 inhibitors have also been clarified that can inhibit the progression of PC [25]. More importantly, PAK1 has been reported to promote gefitinib resistance in lung cancer cells [26]. Ghosh et al. have pointed that PAK1 contributes to tamoxifen resistance in breast cancer [27]. Consistently, our research also unveiled that silencing of PAK1 could improve GEM resistance in PC cells.

In conclusion, our study confirmed that silencing of circLMTK2 could markedly attenuate cell proliferation, invasion/migration, and apoptosis in GEM-resistant PC cells. We also demonstrated that circLMTK2 knockdown inhibited GEM-resistant associated cell proliferation, migration, invasion, and promoted cell apoptosis through the miR-485-5p/PAK1 axis. Furthermore, compared with the control group, the GEM-resistant tumor xenografts of circLMTK2 knockdown group were significantly smaller. Our results suggest that the circLMTK2/miR-485-5p/PAK1 axis may be a potential therapeutic target to improve the sensitivity response of PC to GEM.

## Figures and Tables

**Table 1:** List for primers used for qRT-PCR.

Primer sequence		
circLMTK2	Forward	CGAGGACTGGAAGAAGGAAA
	Reverse	AGGTTTGAATACGGCTGTGC
miR-485-5p	Forward	CGAGAGGCTGGCCGTGAT
	Reverse	AGTGCAGGGTCCGAGGTATT
PAK1	Forward	CAACTCGGGACGTGGCTAC
	Reverse	CAGTATTCCGGGTCAAAGCAT
U6	Forward	CTCGCTTCGGCAGCACA
	Reverse	AACGCTTCACGAATTTGCGT
GAPDH	Forward	CCACATCGCTCAGACACCAT
	Reverse	ACCAGGCGCCCAATACG

## Data Availability

The original data of this manuscript can be obtained from the corresponding author under reasonable request.
